# A DIGE study on the effects of salbutamol on the rat muscle proteome - an exemplar of best practice for data sharing in proteomics

**DOI:** 10.1186/1756-0500-4-86

**Published:** 2011-03-28

**Authors:** Jenna Kenyani, J Alberto Medina-Aunon, Salvador Martinez-Bartolomé, Juan-Pablo Albar, Jonathan M Wastling, Andrew R Jones

**Affiliations:** 1 Institute of Infection and Global Health, University of Liverpool, Crown Street, Liverpool, UK; 2 Spanish Institute for Proteomics (ProteoRed), Centro Nacional de Biotecnología, Consejo Superior de Investigaciones Científicas, Madrid, Spain; 3 Institute of Integrative Biology, University of Liverpool, Biosciences Building, Crown Street, Liverpool, UK

## Abstract

**Background:**

Proteomic techniques allow researchers to perform detailed analyses of cellular states and many studies are published each year, which highlight large numbers of proteins quantified in different samples. However, currently few data sets make it into public databases with sufficient metadata to allow other groups to verify findings, perform data mining or integrate different data sets. The Proteomics Standards Initiative has released a series of "Minimum Information About a Proteomics Experiment" guideline documents (MIAPE modules) and accompanying data exchange formats. This article focuses on proteomic studies based on gel electrophoresis and demonstrates how the corresponding MIAPE modules can be fulfilled and data deposited in public databases, using a new experimental data set as an example.

**Findings:**

We have performed a study of the effects of an anabolic agent (salbutamol) at two different time points on the protein complement of rat skeletal muscle cells, quantified by difference gel electrophoresis. In the DIGE study, a total of 31 non-redundant proteins were identified as being potentially modulated at 24 h post treatment and 110 non redundant proteins at 96 h post-treatment. Several categories of function have been highlighted as strongly enriched, providing candidate proteins for further study. We also use the study as an example of best practice for data deposition.

**Conclusions:**

We have deposited all data sets from this study in public databases for further analysis by the community. We also describe more generally how gel-based protein identification data sets can now be deposited in the PRoteomics IDEntifications database (PRIDE), using a new software tool, the PRIDESpotMapper, which we developed to work in conjunction with the PRIDE Converter application. We also demonstrate how the ProteoRed MIAPE generator tool can be used to create and share a complete and compliant set of MIAPE reports for this experiment and others.

## Introduction

A variety of high-throughput experimental techniques are available for studying how the protein complement of a sample (the proteome) changes under different cellular conditions, such as during disease processes. The changes observed in individual proteins, or groups of proteins, as experimental conditions vary allow researchers to begin understanding the underlying molecular mechanisms in the cell. Gel electrophoresis (GE) has been employed to study proteins for over four decades [1]. GE is frequently applied in two dimensions, whereby proteins are separated by charge followed by molecular weight [2]. More recently, the difference in-gel electrophoresis technique (DIGE) [3] has improved the relative quantification of proteins on 2-D gels. In DIGE, the whole proteomes of different samples are labelled with different fluorescent dyes, mixed and applied to a single gel, thus reducing gel to gel variability in protein migration. Despite the relative age of gel-based proteomic techniques, and recent advances in liquid chromatography-mass spectrometry (LC-MS) for protein quantification, gel-based techniques are still commonly used. For all proteomic techniques, it has been widely documented that the protocols employed can influence the results, for example introducing variability in the set of proteins detected or the estimation of their individual abundances. It is thus important to capture and report a detailed set of information (termed metadata) about how experiments were performed and analysed to allow groups to verify findings, employ similar protocols in their own labs or compare data sets generated in different experiments.

The Human Proteome Organisation - Proteomics Standards Initiative (HUPO-PSI, [4]) was created to help scientists share their data, deposit data sets in public databases and provide tools to assist other groups in performing large scale analysis of public proteomic data sets. In 2007, the PSI published the Minimum Information About a Proteomics Experiment (MIAPE) specification [5]. From this root document, a set of MIAPE modules for proteomics techniques were delivered: gel electrophoresis [6], gel image informatics [7], mass spectrometry [8], mass spectrometry informatics [9], column chromatography [10], capillary electrophoresis [11] and protein-protein or molecular interactions [12]. Each MIAPE module contains a minimal checklist of items that should be reported for the given technique. The items can be reported using plain language, for example describing specific points within the experimental protocols or the data analysis that has been performed, to allow other groups to interpret the published results without ambiguity as to how they were generated. The PSI has also developed data exchange formats, typically represented in Extensible Markup Language (XML). One of these, GelML [13], captures the data related to gel electrophoresis experiments. There are a number of public databases storing protein identification data from proteomics, including PRIDE [14], PeptideAtlas [15], Peptidome [16], the GPMDB [17] and the Swiss2DPAGE database storing GE experiments [18]. However the widely used protein identification repositories (PRIDE, PeptideAtlas etc) are primarily focussed on LC-MS studies and historically have either no GE data sets or no simple mechanism for deposition of data derived from gel-based experiments.

In this article we demonstrate how MIAPE GE (gel electrophoresis) and GI (gel informatics) compliant reports can be created easily in practice, through the MIAPE Generator tool [19], developed by ProteoRed - the Spanish network for proteomics. We have also developed a new tool, the PRIDESpotMapper, to work alongside the PRIDE Converter software [20] to enable GE studies to be captured in the PRIDE XML format and be submitted to the public PRIDE repository. The provision of both the MIAPE report and the public PRIDE record, enables other groups to download the complete data sets, including raw gel images, mass spectra and protein identifications, along with complete descriptions of the experimental protocols.

We have performed a study on the effects of salbutamol (an anabolic agent) on the proteome of rat muscle cells. Salbutamol is a type of beta_2 _adrenergic agonist, which is known to cause hypertrophy in muscle but the underlying molecular mechanisms are not well understood. The aims of the study are to use proteomic technologies to model changes in the development of skeletal muscle cells *in vitro *in the presence of salbutamol and to identify novel proteins and pathways within these cells that interact with these agents, and therefore could be potential targets for their action. DIGE was used to compare control and treated samples at 24 h and 96 h after addition of salbutamol. Gel spots with changed abundance were subjected to tandem mass spectrometry for protein identification. Bioinformatics analysis was performed using the Gene Ontology (GO) [21] and the DAVID tool [22] for determining categories of functions that appear to be enriched at the different time points.

In the supplementary material [Additional file 1], we include the protocols employed in the DIGE study, as they would be reported in a standard journal article. We have also used the ProteoRed MIAPE Generator to create MIAPE GE and GI compliant reports (described in [19]) and we use these examples to demonstrate how a standard set of materials and methods map into the MIAPE reports generated, to act as a practical guide to MIAPE for proteome scientists. We have also deposited the MS data sets and identifications in PRIDE, using the PRIDESpotMapper and PRIDE Converter, for public access and review.

### Software development

The PRIDE Converter software [20] enables conversion from a variety of mass spectra and search engine file formats into the PRIDE XML format that can subsequently be used for uploading spectra and peptide/protein identifications into the PRIDE database. However, the PRIDE Converter has been designed primarily for "shotgun proteomics" experimental designs, where peptide to protein inference is performed across all input spectra, which is not well suited to gel-based studies. The software is capable of loading multiple identification files (e.g. Mascot dat files or Sequest .out files), but in its internal processing, the resulting proteins are inferred from a combined list containing all the identified peptides. For gel-based studies, typically each identification file (say one Mascot dat file) comes from a single gel spot and its identified peptides should not be combined with those from other spots. The PRIDE Converter also has no mechanism for uploading gel image coordinates, or additional information regarding protein quantification. To overcome these limitations, a custom version of the PRIDE Converter was developed by the PRIDE team, where every identified peptide was annotated with the name of the source gel spot. Simultaneously, we developed a new application called "the PRIDESpotMapper" as a complement to the PRIDE Converter for gel-based experiments. This was implemented in Java and modifies the PRIDE XML file generated using the custom PRIDE Converter, dividing the identified proteins according to the source identification file for each gel spot. Starting from a PRIDE XML file and either an XML or Excel spot map (see [Additional File 2] for the format specifications) the application ensures that records are created for each identified protein, derived from peptide identifications from each input file independently.

**Figure 1:**
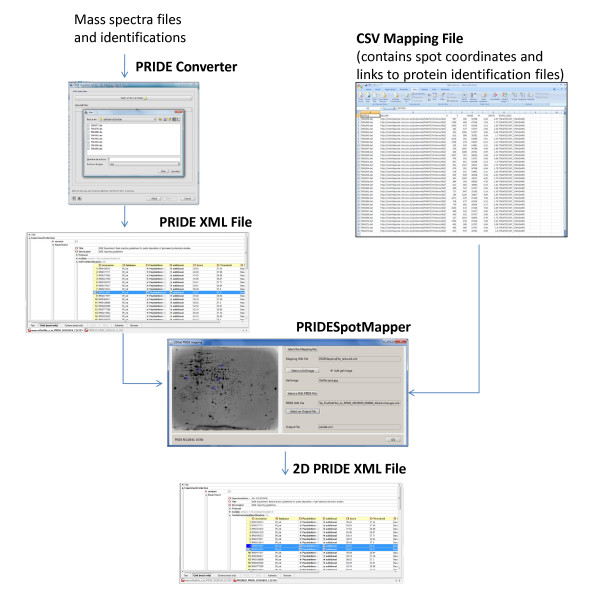
**The workflow used to construct a PRIDE XML file containing gel spot data, via the modified PRIDE Converter and the PRIDESpotMapper**.

Once all the resulting files coming from the search engine (Mascot for this version) are joined in a single PRIDE XML using the PRIDE Converter, the execution of the PRIDESpotMapper is straightforward (Figure 1). First, either the XML or Excel spot map file should be entered. Second, the gel image can be loaded from a local file or from a URI, for example if gel images have been loaded into the ProteoRed MIAPE Generator database [19]. Third, the previously created PRIDE XML file is required. The application merges the two data files (Spot map file and PRIDE XML file) to create a new PRIDE XML file (internally called 2D PRIDE XML file), in which each spot is linked to one protein only with its corresponding peptides, alongside gel spot coordinates and relative quantification data. The file is then saved on the local drive, ready for upload to the PRIDE database.

## Results

The DIGE gels were analyzed as described in the Supplementary Methods [Additional file 1] and sets of spots were identified as being differentially expressed at the 24 h time point (versus the untreated control) and 96 h time point (versus control). The protein(s) contained within those spots were then identified by tandem mass spectrometry (MS/MS). In most spots, more than one protein was identified, indicating that some co-migration of proteins occurred (and the high sensitivity of MS/MS). As such it is not always possible to link exact quantitative differences between conditions to specific proteins, although general conclusions can be made about the groups of proteins that have changed between conditions. The proteins that were identified with high confidence were further analyzed using the DAVID tool [22], which highlighted several functions that were strongly enriched (discussed below).

At the 24 h time point 17 spots of interest were identified: 4 spots were down-regulated, 13 spots up-regulated from which 31 non-redundant proteins were identified [Additional file 3]. 23% of the proteins identified are cytoskeletal and also are mapped in pathways involved with skeletal development (based on Gene Ontology terms). At the 96 h time point 35 spots of interest were identified - 11 spots were down-regulated, 24 spots up-regulated [Additional file 4]. From these spots 110 non-redundant proteins were identified. 25% of these proteins are cytoskeletal proteins. Several of these proteins, e.g. vimentin and desmin, are known to be involved in the skeletal development pathway. There are several proteins modulated in both sample sets, such as beta-enolase that is involved in glycolysis. Some proteins were found in more than one spot, which could suggest the presence of post-translational modifications, such as phosphorylation.

We have performed gene ontology enrichment analysis on the two data sets using DAVID (24 h [Additional file 5] and 96 h [Additional file 6]). At 24 h the main functional clusters enriched were: "contractile fiber", "cytoskeleton", "calcium ion binding" and "collagen biosynthetic process". At 96 h post treatment, the main enriched functional categories were "cytoskeleton", "tubulin", "microtubule-based movement", "GTPase activity" "cellular protein complex assembly", "contractile fiber" and "regulation of ATPase activity" amongst several others.

In summary, proteins involved in ion binding and transport, nucleosome assembly, cell interactions, protein binding and structural proteins seem to be modulated at 96 h, whereas only structural and energy production proteins are affected after 24 h. An immediate effect of the anabolic agent is to produce a structural effect that needs a great deal of energy. It appears a more complex later effect is observed, involving a number of cellular pathways.

Salbutamol is shown to cause muscle hypertrophy, which suggests it may have a similar mode of action as other beta_2_-adrenergic agonists. This effect on the muscle cell is rapid, and is clearly seen using high magnification microscopy. As anticipated, many of the differentially expressed proteins identified are cytoskeletal. A significant number are also involved in transcription or translation. Skeletal development pathways are activated both at the early and later time points. Up-regulation of ATP synthesis, glycolysis and phosphorylation also seems to be occurring.

### Data deposition and MIAPE report generation

MIAPE guideline documents describe the metadata that should be captured about a given proteomic technique, for instance detailing the minimum information that should be reported about the experimental protocols. The MIAPE Generator tool has been developed to assist in the generation of MIAPE-compliant reports, and is freely accessible from http://www.proteored.org/. The tool guides users through each stage of the report creation process, capturing all details required by the underlying MIAPE module. The tool's user interface is based on a series of web forms for data entry, built on top of a relational database. These forms follow a hierarchical structure according to the original sections of each MIAPE module. Each document is always linked to a project, which can be viewed and accessed only by the project owner, until the document is ready for public access. The tool has a template system such that protocols can be re-used in different reports to avoid repetition in data entry, and drop-down boxes are provided as applicable, containing controlled vocabulary or ontology terms to capture standard terminology for techniques, units and so on, to allow reports to be compared automatically. The generated reports are stored in the database and can be exported in a variety of formats. In this instance, the MIAPE Generator tool was used to create two reports for each time point, capturing the methods detailed above concerning gel electrophoresis sections (in the MIAPE GE report) and concerning the gel image informatics sections (in the MIAPE GI report) - see "Availability and requirements".

The benefits of producing the MIAPE reports, in addition or instead of a traditional Materials and Methods, are as follows. The report has a standard structure, requesting key details for each stage of the process, ensuring that the experimenter does not fail to report any information that may be important for reproducing the protocols in another lab. As one example, the MIAPE GE document requests that the gel recipe are provided (section 3.2.2) if the gel was not purchased pre-cast. Similarly, the MIAPE GI document requests details should be provided on software parameters and algorithms used with different software packages, which can affect the results obtained. The MIAPE GE/GI specifications also request that raw data should be provided and linked to the report, in this case, the original gel images. This could potentially be hugely valuable if researchers are interested in performing more detailed examination of results, for example to test if a specific protein is differentially regulated, using different statistical assumptions than the researchers who generated the data.

### Deposition of protein identification data in PRIDE

The PRIDE database has become one of the leading public repositories for proteomics results. However, to date, few gel studies have been deposited in PRIDE due to the lack of appropriate tools. We have deposited two files - one for the 24 h and one for 96 h time point, each containing the protein identifications for each gel spot. Each protein identification has a link to the gel image in the MIAPE database, along with X/Y coordinates and quantification information, in terms of the ratio detected by DIGE across treated versus control samples. As far as we are aware, this is the first deposition of a complete quantitative DIGE data set in PRIDE. The PRIDE records can be accessed at http://www.ebi.ac.uk/pride/ under accessions 16472 and 16473. Data files download from PRIDE can be visualised using the PRIDEViewer software [23].

## Discussion and Conclusions

Each year there are many hundreds of proteomics studies published in the literature, in which gel electrophoresis is used to separate, identify and perform relative quantification of the proteins present in complex samples. However, few of these data sets have ever made it into the public domain, beyond lists of protein spots provided in tables within articles or as supplementary material in spreadsheets. The Proteomics Standards Initiative has released several tools and guideline documents designed to improve the public accessibility of proteomics data, including minimum reporting guidelines (MIAPE documents) and XML formats. The EBI has also developed the PRIDE database to allow proteomics scientists to publish protein identification data sets to the wider community.

While it is possible to include protein identification data in the database behind the MIAPE generator tool, this is not the standard public repository for this kind of data. Instead, proteomics scientists tend to search the PRIDE database (or GPM, PeptideAtlas, Peptidome, Tranche) for identification data. As such, it is important for gel-based proteomics studies to be deposited in one of these primary data repositories. To date, almost no data sets derived from gel-based experiments have been deposited in any of these databases. We have created the PRIDESpotMapper to work alongside the PRIDE Converter and thus, for the first time, provide a simple route for uploading valid PRIDE XML, containing gel spot information and quantitative values. The PRIDE developers may include gel support in future versions of the PRIDE Converter directly. We will work with the PRIDE team to incorporate the same mechanism presented here for representing gel spot data, to ensure that researchers wishing to share gel-based proteomic data can use the PRIDESpotMapper now, and migrate to a new version of the PRIDE Converter, as and when appropriate.

This article should serve as an exemplar for how researchers can upload gel-based data to PRIDE and use the MIAPE Generator tool for creating MIAPE compliant reports. There is on-going discussion with journal editors as to the requirement for proteomics articles to be MIAPE compliant - and these reports may in due course supplement, or in some cases replace, traditional materials and methods sections of proteomics articles. We encourage further discussion of these issues, for example through the PSI's open mailing lists or attendance at the annual PSI meeting.

For the study described, we have created MIAPE reports describing the gel electrophoresis and the gel image informatics performed, and these have been deposited in the associated database. The database also contains the source gel images, allowing other groups to re-analyse these data, using the same or different software pipelines.

The initial results of the study show that several key pathways are modulated by treatment with salbutamol, with significantly more changes occurring at 96 h post-treatment. This indicates that there is a lag between the treatment and the downstream activation of cellular pathways. There are some limitations of the DIGE results, not least that true quantitative ratios cannot be linked to individual protein identities, since the sensitivity of tandem MS revealed that many spots on the gels contained more than one protein. However, the ontology enrichment analysis shows that many of the protein groups highlighted are likely to be direct or indirect targets for salbutamol, since the enriched functional categories fit our expected hypotheses of the effects of an adrenergic agonist. We are making these data sets freely available as we anticipate that they will be useful to other researchers working in this area for building hypotheses about the mechanism of action of salbutamol on the proteome of muscle tissue.

## Availability and requirements

The software described in this article is accessible from http://proteo.cnb.csic.es/pridespotmapper.

The adapted PRIDE Converter described here is released as a snapshot build (2.4.2), which will be updated periodically when there are major new releases of the main PRIDE Converter software but not for minor updates. The PRIDE Converter is freely available and open-source, released under Apache License 2.0. PRIDE Converter requires Java 1.5 (or above). The current version has been tested on Windows XP, Windows Vista, Linux and Mac OS X.

The PRIDESpotMapper is freely available as a Java jar file for local install or can be run using Java web start. The application has tested using Java Runtime Environment (JRE) 1.6 with the following operating systems: Windows 7, Windows XP, Windows Vista, Linux Red Hat, Linux Ubuntu.

URLs to link direct to these records in the MIAPE Generator database.

24 h timepoint:

http://estrellapolar.cnb.csic.es/proteored/MIAPE/MIAPE_GE.asp?pmCodigoAcceso=415db6c1&pmIDUsuario=2378&pmId=1082

http://estrellapolar.cnb.csic.es/proteored/MIAPE/MIAPE_GI.asp?pmCodigoAcceso=1d9f04d3&pmIDUsuario=2378&pmId=768

96 h timepoint:

http://estrellapolar.cnb.csic.es/proteored/MIAPE/MIAPE_GE.asp?pmCodigoAcceso=ae31268d&pmIDUsuario=2378&pmId=663

http://estrellapolar.cnb.csic.es/proteored/MIAPE/MIAPE_GI.asp?pmCodigoAcceso=a43637ec&pmIDUsuario=2378&pmId=397

PRIDE access for data sets

Accessions 16472 and 16473

Mascot dat files located on Tranche:

24 h time point:

https://proteomecommons.org/dataset.jsp?id=ziJZS3iGNcd5eMDW3vPpCb5VXJ4oorFWe1xwdIaE97hUxDNcXwtXaf6twotWtsTds4RVu84Obfgw2oLp3k7tRjWXWx8AAAAAAAAChw%3D%3D

96 h time point:

https://proteomecommons.org/dataset.jsp?id=bZv347BIF4uVOWlwKok4ASHz2OCgPSXwqxfNP4LB2Qqna6tEnYVQNilPsrlQMgIAZBUAxyJCBpCK2kRqq%2BPCoQIv6oAAAAAAAAACjg%3D%3D

Passphrase: ratproteome

## List of abbreviations

DIGE: difference in-gel electrophoresis; GE: Gel electrophoresis; GI: gel informatics; GO: Gene ontology; HUPO: Human Proteome Organisation; LC-MS: Liquid Chromatography-Mass Spectrometry; MIAPE: Minimum Information About a Proteomics Experiment; PRIDE: PRoteomics IDEntifications database; PSI: Proteomics Standards Initiative; XML: Extensible Markup Language

## Competing interests

The authors declare that they have no competing interests.

## Authors' contributions

JK performed the DIGE study, supervised by JMW. JAMA led the software development work described, assisted by SMB and supervised by JPA. ARJ led the writing of the manuscript and contributed to software development and to analysis of the DIGE results. All authors have contributed text to the manuscript.

## Supplementary Material

Additional file 1 **Supplementary Methods**. The materials and methods from this study Click here for file

Additional file 2 **Example mapping file for the PRIDESpotMapper**. The csv file can be opened, for example, in spreadsheet software. The file contains information for mapping Mascot dat files to spot coordinates on gel images, and is used to create the final PRIDE XML file. Click here for file

Additional file 3 **Proteins with changed abundance at 24 h post-treatment**. A summary file containing the set of proteins that had modulated abundance, as detected by DIGE, at 24 h post-treatment with salbutamol. Click here for file

Additional file 4 **Proteins with changed abundance at 96 h post-treatment**. A summary file containing the set of proteins that had modulated abundance, as detected by DIGE, at 96 h post-treatment with salbutamol. Click here for file

Additional file 5 **Clusters of functional annotations for proteins at 24 h post-treatment**. The output of clustering functional annotations produced by DAVID, on the protein set at 24 h post-treatment Click here for file

Additional file 6 **Clusters of functional annotations for proteins at 96 h post-treatment**. The output of clustering functional annotations produced by DAVID, on the protein set at 96 h post-treatment Click here for file
