# Supplementary material for: A DIGE study on the effects of salbutamol on the rat muscle proteome - an exemplar of best practice for data sharing in proteomics
Source: BMC Res Notes. 2011 Mar 28;4:86. doi: 10.1186/1756-0500-4-86 (PMC3080311; doi:10.1186/1756-0500-4-86)
Supplement: Additional file 1 — Supplementary Methods. The materials and methods from this study [file 1756-0500-4-86-S1.DOC]

**Supplementary Methods**

*Cell culture*

Primary rat skeletal muscle cells were prepared by Pfizer, which were transferred into cell growth medium (DMEM, 20% FCS, 1.4% antibiotic-Antimycotic solution). After three days of incubation, when cell layers were near or fully confluent, treatment occurred. First each well was washed with 5 ml PBS (137 mM NaCl, 2.7 mM KCl, 10 mM Na_2_HPO_4_, 2 mM KH_2_PO_4_, pH 7.2). Following this, 4.5 ml of cell fusion medium (2.5% DHS, DMEM and Antibiotic-Antimycotic Solution) was added to each well. On each six well plate 500 µl 10^-4^ M salbutamol, dissolved firstly in Dimethyl sulfoxide (DMSO) then added to a DMEM solution, was used to treat three of the wells. The other three wells were used as control wells and had 500 µl 1% DMSO in DMEM solution added. Cells were incubated for different lengths of time: 24 h and 96 h. For each time point six plates were collected, images taken and then wells washed with PBS three times and frozen at -80°C.

*Sample Preparation*

One ml of lysis buffer (30 mM Tris-Cl, 2 M Thiourea, 7 M Urea, 4% CHAPS) was added to each well and scraped thoroughly before being transferred to a sterilized eppendorf tube. Samples were treated to a freeze/thaw process four times, and then centrifuged at 13000 rpm for 10 min and the pellet discarded. Precipitation of the protein was achieved by adding 5 volumes of ice cold acetone to the samples and incubating them at -20°C overnight. Samples were centrifuged at 5000 rpm for 5 min at 4° C before carefully removing the supernatant and leaving the pellets to air dry. Protein concentration was determined using the BCA assay (Bio-Rad Protein Assay). Four biological replicates were collected.

*CyDye Labelling*

After acetone precipitation the pellet was resolubilised in lysis buffer to give a final concentration of 5 µg/µl. The pH level was checked to ensure it was at 8. The rat cell lysates were labelled using CyDye DIGE Fluor minimal dyes, according to the manufacturer’s recommended protocols (GE Healthcare). 50 µg of the control sample and the treated sample were added to 1 µl (400 ρmols/μl) of working Cy3 or Cy5 (depending on the sample) and mixed by vortexing. Control and treated samples were alternately labelled with either Cy3 or Cy5 to normalize for dye specific effects. Equal amounts of all samples were mixed together to produce an internal standard to minimise differences caused by sample preparation, which was labelled with Cy2. The labelled mixtures were incubated on ice in the dark for 30 min and the reaction was terminated by addition of 1 µl of 10 mM lysine. The two samples plus the internal pool were mixed prior to IEF. Equal volumes of 2 x sample buffer (7 M Urea, 2 M Thiourea, 4% (w/v) CHAPS) were added to each of the four biological replicates. The volume of the samples was made up to 450 μl using rehydration buffer (7 M Urea, 2 M Thiourea, 4% (w/v) CHAPS, 2% (w/v) DTT and 2% ampholytes).

*2D-DIGE*

Immobiline DryStrips, pH 3-10 Non-Linear 24 cm (GE healthcare), were rehydrated over night with the rehydration sample mix using a reswelling tray (GE healthcare). IEF was carried out on an Ettan^TM^ IPGphor II^TM^ (GE healthcare) following the manufacturer’s handbook. Strips were focused at 50 µA per strip following a three step protocol involving: 3 h step at 300 V, 3 h Gradient at 600 V, 3 h Gradient at 1000 V.

Prior to the second dimension the strips were equilibrated in SDS-equilibration buffer (2% SDS, 50 mM Tris-HCL pH 8.8, 6M urea, 30% (v/v) glycerol and 0.002% (w/v) bromophenol blue) twice for 15 min. DTT 10 mg/ml was present in the first equilibration and 25 mg/ml of idoacetamide was included in the second round. The strips were immediately applied to a 12.5% SDS-PAGE gel cast using the protocol in the Ettan DIGE System manual (GE healthcare). Low-fluorescence Glass Plates, 27 × 21 cm (GE Healthcare) were used in casting of the gels. The back plates were treated with bind-silane solution (80% (v/v) EtOH, 18% (v/v) dH2O, 2% (v/v) acetic acid, 0.1% (v/v) γ-methacryloxypropyltrimethoxysilane). Reference markers were placed on the back plates before gels were cast. Gels were run in the Ettan DALT*six* System which was assembled as guided by the handbook. The gels were run for 30 min at 5 W per gel and then 4 h at 17 W per gel or until the tracking dye reached the bottom edge.

A preparative gel was prepared using 400 µg of a pooled sample made from all biological replicates of treated and untreated samples. This gel was run under the same conditions as the previous gels and at the same time. The gel was fixed in 40% (v/v) MeOH, 10% (v/v) acetic acid for one hour then washed twice with ddH_2_O for 10 min before staining with SYPRO® Ruby gel stain (Invitrogen) overnight. The gel was then washed in 10% (v/v) MeOH, 7% (v/v) acetic acid for one hour followed by two 5 min washes in ddH_2_O.

*Image acquisition*

All gels were scanned using Ettan^TM^ DIGE Imager (GE Healthcare) following the manufacturer’s guidelines. Cy3 gel images were scanned at an excitation wavelength of 540/25 nm (maxima/bandwidth) and an emission wavelength of 595/25 nm, while the Cy5 images were scanned at an excitation wavelength of 635/30 nm and an emission wavelength of 680/30 nm. The internal standards images were scanned at an excitation wavelength of 480/30 nm and an emission wavelength of 530/40 nm. For the preparative gel the Imager was set to Sypro scanning and scanned at an excitation wavelength of 540/25 nm and an emission wavelength of 595/25 nm.

*Image analysis*

DeCyder^TM^ Differential Analysis Software (GE Healthcare) was used to analyse the gel images to allow spots of interest to be identified. Twelve gel images of three dye channels from four gels were uploaded into the software. Every spot on each gel was identified and a comparison from across the image channels (Cy2/Cy3/Cy5) on the same gel was made. To gain statistical data on the protein expression levels of each spot, a comparison of all images from the four different gels was made, allowing modulated proteins to be identified. Any protein spots which appeared in nine of the twelve images and had a +/- fold change of 1.5 or above (p<0.05) was highlighted as a spot of interest. The preparative gel image was matched to the DIGE gels and any spots of interest were identified on this gel before being picked.

*Trypsin digestion*

Trypsin digestion was performed on all spots individually. The spots were destained by incubating at 37°C with 50 mM ammonium bicarbonate (Ambic) /50% Acetonitrile (ACN) for 10 min and then the destain process repeated. Following this, 10 µl of 100% ACN was added to each spot and again incubated at 37°C for 15 min, at which point the plug turned white. The solvent was removed and samples replaced into an incubator for 10 min to allow the remaining solvent to evaporate. Sequence grade trypsin (Roche) was diluted with 50 mM Acetic acid to give a stock solution of 100 ng/µl. This was then diluted 1/10 with 25 mM Ambic, 10 µl of this solution was added to each well. These were then incubated for 30 min to 1 h at 37°C. A further 10 µl of 25mM Ambic was combined with the solution and left to incubate at 37°C overnight. To stop the reaction 2 µl of 2.6 M formic acid was applied and then the samples were stored at -20°C.

*Mass spectrometry*

The LTQ (LC-MS/MS) (Thermo Fisher Scientific) was used to acquire spectra for all spots. Briefly, the LTQ uses a Dionex Ultimate 3000 HPLC system equipped with a nano C18 Pepmap reversed phase column. An injection of 10 µl of the tryptic peptides, diluted in 1% Formic acid, was loaded on to a C18 TRAP, desalted and washed before being transferred on to the column. The peptides were eluted over a continuous linear gradient of 0-50% acetonitrile. The ionised peptides were analysed using the data-dependant “triple play” mode. After the initial MS scan a charge state is allocated to each ion in the “zoom scan” mode. The threshold for peptides to be further analysed was set at 20,000. The three most intense ions above the set threshold are selected to be fragmented and subjected to a final MS/MS scan.

Once spectra (dta files) were collected for samples these were merged into mgf files before submitting to MASCOT. The search was set to use the rat IPI database (v 3.53) with parameters: fixed modification of carbamidomethyl on cysteine residues, variable oxidation modification on methionine, peptide tolerance of +/- 1.5 Da, MS/MS tolerance of +/- 0.5 Da and a maximum of 1 missed cleavage.
